# Supplementary material for: Transconjunctival versus Transcutaneous Injection of Botulinum Toxin into the Lacrimal Gland to Reduce Lacrimal Production: A Randomized Controlled Trial
Source: Toxins (Basel). 2021 Jan 21;13(2):77. doi: 10.3390/toxins13020077 (PMC7911887; doi:10.3390/toxins13020077)
Supplement: Supplementary file 1 [file toxins-13-00077-s001.pdf]

Supplementary Materials: Transconjunctival versus Transcutaneous Injection of Botulinum Toxin into the Lacrimal Gland to Reduce Lacrimal Production: a Randomized Controlled Trial

Andrew G. Lee, Shin-Hyo Lee, Minsu Jang, Sang Jae Lee and Hyun Jin Shin

Table S1. Findings of objective and subjective evaluations after the botulinum toxin BTX-A injections into the lacrimal. GBI, Glasgow Benefit Inventory. Data are *n* (%) or mean ± standard-deviation values.

Variable	CON Group	CUT Group	*p*
TMH, mm			
2 weeks after intervention	0.22 ± 0.07	0.27 ± 0.07	
6 weeks after intervention	0.20 ± 0.08	0.24 ± 0.10	
12 weeks after intervention	0.22 ± 0.07	0.25 ± 0.09	
24 weeks after intervention	0.22 ± 0.07	0.23 ± 0.08	0.218 [a]
STA value, mm			
2 weeks after intervention	11.11 ± 6.62	10.44 ± 7.11	
6 weeks after intervention	9.20 ± 5.98	11.07 ± 7.59	
12 weeks after intervention	11.29 ± 5.36	11.24 ± 6.50	
24 weeks after intervention	11.44 ± 6.15	9.32 ± 5.14	0.459 [a]
Outdoor Munk score			
2 weeks after intervention	1.79 ± 1.17	1.44 ± 0.70	
6 weeks after intervention	1.46 ± 0.92	1.33 ± 0.62	
12 weeks after intervention	1.14 ± 1.07	1.08 ± 0.95	
24 weeks after intervention	1.25 ± 0.97	1.48 ± 1.19	0.218 [a]
Indoor Munk score			
2 weeks after intervention	1.14 ± 1.15	0.85 ± 0.82	
6 weeks after intervention	0.89 ± 0.92	0.59 ± 0.57	
12 weeks after intervention	0.79 ± 0.83	0.44 ± 0.51	
24 weeks after intervention	0.71 ± 0.66	0.72 ± 0.89	0.082 [a]
GBI score			
6 weeks after intervention	14.23 ± 21.32	13.70 ± 20.37	
12 weeks after intervention	18.58 ± 16.69	12.59 ± 12.64	

24 weeks after intervention	15.63 ± 14.80	12.96 ± 12.80	0.415 [a]
Duration of symptom relief, months	5.25 ± 2.42	6.07 ± 1.48	0.176 [b]
Adverse effect	3/28 (10.7%)	3/25 (12%)	1.000 [c]
Recommendation to others	21/28 (75%)	18/25 (72%)	1.000 [c]
Overall success	25/28 (89.3%)	21/25 (84%)	0.694 [c]

CON, transconjunctival injection; CUT, transcutaneous injection; [a] Repeated-measures analysis of variance; [b] Mann-Whitney *U* test; [c] Fisher's exact test.

Table S2. Change in Glasgow Benefit Inventory (GBI) of enrolled patients on a 5 point Likert scale: 1 = Much worse, 2 = A little or somewhat worse, 3 = No change, 4 = A little or somewhat better, 5 = Much better.

	Question	Score 6 Weeks	Score 12 Weeks	Score 24 Weeks	Average Score
1.	Has the result of the operation affected the things you do?	3.6 ± 0.7	3.5 ± 0.7	3.7 ± 0.6	3.57
2.	Have the results of the operation made your overall life better or worse?	3.6 ± 0.9	3.8 ± 0.7	3.6 ± 0.7	3.65
3.	Since the operation, have you felt more or less optimistic about the future?	3.6 ± 0.8	3.8 ± 0.6	3.6 ± 0.6	3.65
4.	Since your operation, do you feel more or less embarrassed when with a group of people?	3.2 ± 0.7	3.3 ± 0.7	3.1 ± 0.7	3.22
5.	Since your operation, do you have more or less self-confidence?	3.5 ± 0.8	3.4 ± 0.7	3.6 ± 0.6	3.50
6.	Since your operation, have you found it easier or harder to deal with company?	3.4 ± 0.8	3.4 ± 0.6	3.4 ± 0.6	3.41
7.	Since your operation, do you feel that you have more or less support from your friends?	3.1 ± 0.6	3.3 ± 0.5	3.3 ± 0.5	3.23
8.	Have you been to your family doctor, for any reason, more or less often, since your operation?	2.7 ± 0.5	2.9 ± 0.5	2.7 ± 0.7	2.80
9.	Since your operation, do you feel more or less confident about job opportunities?	3.1 ± 0.3	3.1 ± 0.4	3.2 ± 0.5	3.15
10	Since your operation, do you feel more or less self-conscious?	2.8 ± 1.0	2.7 ± 0.9	2.7 ± 0.7	2.73
11	Since your operation, are the more or fewer people who really care about you?	3.2 ± 0.7	3.2 ± 0.7	3.3 ± 0.6	3.23
12.	Since you had the operation, do you catch colds or infections more or less often?	3.5 ± 0.9	3.6 ± 1.1	3.6 ± 0.9	3.55
13	Have you had to take more or less medicine for any reason, since your operation?	2.9 ± 0.2	3.0 ± 0.2	2.9 ± 0.6	2.94
14	Since your operation, do you feel better or worse about yourself?	3.7 ± 0.9	3.7 ± 0.8	3.6 ± 0.7	3.63
15	Since your operation, do you feel that you have had more or less support from your family?	3.4 ± 0.9	3.4 ± 0.6	3.4 ± 0.8	3.39
16	Since your operation, are you more or less inconvenienced by your health problem?	3.5 ± 1.1	3.5 ± 1.0	3.5 ± 0.8	3.52
17	Since your operation, have you been able to participate in more or fewer social activities?	3.1 ± 0.3	3.1 ± 0.4	3.1 ± 0.3	3.07
18	Since your operation, have you been more or less inclined to withdraw from social situations?	3.1 ± 0.4	3.0 ± 0.3	3.0 ± 0.3	3.02
	Rasch scale (score, −100–100)*	14.0 ± 20.4	15.7 ± 14.8	14.3 ± 13.6	14.65

*A final score is calculated using a Rasch scale, which ranges from −100 (maximum detriment) to 100 (maximum benefit).

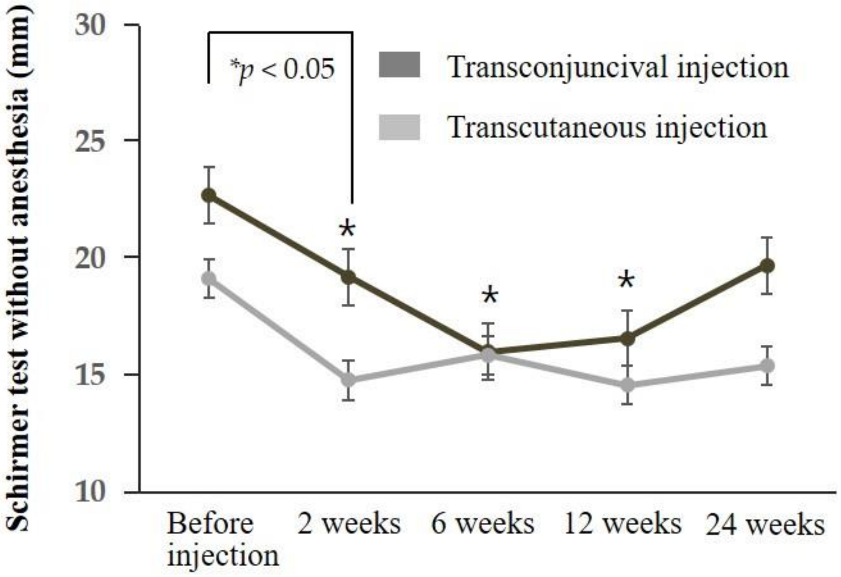

Figure S1. Change in value of Schirmer's I test without topical anesthesia during the 24 weeks of follow-up in the CON and CUT groups. The STA value decreased significantly relative to before injections ($p < 0.05$) at 2, 6, and 12 weeks. *RMANOVA.
